# Complexities and Questions Toward Artificial Intelligence for Diagnostic Support in Virtual Primary Care

**DOI:** 10.1016/j.mcpdig.2023.10.005

**Published:** 2023-11-24

**Authors:** Jacqueline K. Kueper

**Affiliations:** Institute for Better Health, Trillium Health Partners, Mississauga, and Department of Epidemiology and Biostatistics, Schulich School of Medicine and Dentistry, Western University, London, Ontario, Canada

The challenge of effectively deploying artificial intelligence (AI) technologies into real-world settings is understudied for primary care, which has received less focus than other health care sectors in the AI revolution.^1^ Zeltzer et al,^2^ address this gap with their study published in Mayo Clinic Proceedings: Digital Health on 102,059 AI-generated diagnoses in virtual primary care encounters through the K Health platform in the United States from October 2022 to January 2023.^2^ Their research suggests potential roles for AI to support virtual primary care, and showcases diagnostic challenges and sociotechnical complexities amidst the broad scope of primary care. This editorial focuses on 4 areas with the intention to motivate future work: heterogeneous presentations and conditions, data sources and quality, human-AI interaction, and social queries.

### Heterogeneous Presentations and Conditions

Diagnostic challenges in primary care are first exemplified by Zeltzer et al's^2^ methodological decision to exclude from analyses the 8.4% (n=9946) otherwise eligible patients who completed the AI chat-based intake procedure and proceeded to see a human provider that assigned a final nonspecific International Classification of Diseases (ICD)-10 code, and the 4.9% (n=5790) who received more than 1 code (diagnosis). Accurate diagnosis to guide appropriate treatment or follow-up care is important for these cases; mixed methods research may be useful to investigate the role of AI in identifying these messier end points.

Within the analytic sample, subgroup analyses by final provider-assigned diagnosis echo the sentiment that rare conditions are common in primary care.^3^ Although results for NIH-defined rare diseases are not available, agreement rates between providers and AI differential diagnoses among less common conditions often fell below performance for the most common conditions and presenting complaints. Of the 992 diagnoses identified, 8 had over 3% prevalence, accounting for 69.4% (n=70,788) of the analytic sample.^2^ Of the remaining less common provider-assigned diagnoses, 71 were reported across 27.7% (n=28,225) of the analytic sample: 31 (43.7%) were in the AI differential diagnosis list less than 75% of the time and 12 (16.9%) were never suggested by the AI model.^2^ These lower agreement rates occurred for 14.9% (n=15,256) to 17.9% (n=18,302) of the analytic sample, depending on how many of the privacy restricted small cell counts follow the same low accuracy pattern.^2^ It is also noteworthy that the diagnoses with the highest agreement rates (eg, urinary tract infection) are generally easier for human providers to diagnose than those with lower agreement rates (eg, asthma). A deeper investigation into the AI model’s performance for atypical presentations of common conditions would also be interesting.

The variable performance across diagnoses highlights the importance of clearly communicating intended uses and limitations of an AI model to end-users and the importance of training and education on how to incorporate AI suggestions into clinical decision-making.^4^ The presentation of model information should be tailored to each type of end-user. For example, both patients and providers are end-users throughout the AI-first care paradigm at K Health.^2^

### Data Sources and Quality

AI model performance relies on available input data, and there are pros and cons to different data types for any given task. Cross-sectional patient interview data, traditionally gathered by a provider and obtained by an AI-based chat at K Health, has the benefit of detailed patient-provided information at the time of the clinical encounter. Data quality depends on the person’s ability to describe their symptoms and historical risk factors, and on the interview technique and interpretation of responses.^5^ Information biases in self-reported data are well known, such as recall bias in recounting past experiences and the influence of one’s emotional state and context on symptom reporting.^5^ Longitudinal electronic health record data, which many AI diagnostic models are trained with including the baseline models in Zeltzer et al,^2^ mitigate some information biases, but are susceptible to their own quality and completeness challenges.^6^ Prospective medical tests, which may be ordered if a diagnosis cannot be made on the basis of the first 2 sources, can be high quality but are not always necessary and have time and resource costs. The most valuable data type for any given diagnosis will depend on the person and condition. In pursuit of a general purpose primary care diagnostic model, multimodal techniques may be useful and could additionally incorporate imaging data and other everyday data sources such as wearables, noting quality and validation of these other sources will also be crucial.

The measure of diagnostic accuracy itself is limited by data quality of a gold standard label. Zeltzer et al^2^ measure accuracy as agreement between the AI differential diagnosis and final provider selected ICD-10 code. The secondary adjudication analyses showcases the inter-provider variance of diagnosis in primary care (58.2% consensus),^2^ and biases in health care are known to be prevalent.^7^ Biases result in differential care quality across population subgroups, including diagnostic inaccuracies, which end up being encoded in health records.^7^ Obtaining large numbers of unbiased, reliable, and valid diagnoses for training or validation is often impractical because of resource and human constraints. Diagnostic accuracy studies, including subgroup analyses, could be supplemented by performance assessments in terms of important patient outcomes, such as success of prescribed treatments or need for further diagnostic inquiries.

### Human-AI Interaction

Across the analytic sample, providers selected the top AI prediction 60.9% of the time, otherwise selecting a condition lower in the AI differential diagnosis (23.3%) or not there at all (15.8%).^2^ Zeltzer et al^2^ discuss their findings in terms of potential roles for AI to serve as a diagnostic or triage tool to optimize provider workflow and system efficiency—a formal evaluation of time and other clinical workflow impacts would be a welcome addition to the research literature, as would an evaluation of diagnostic understanding and outcomes for people who viewed the AI differential diagnosis after their AI-based intake interview and decided to not continue on to a human provider appointment and final diagnosis.

The effectiveness of AI to support triage and diagnosis depends on factors beyond model performance, such as user interface design and how and when results are viewed. A study on AI support for knee magnetic resonance imaging and electrocardiogram interpretation found that showing AI recommendations to providers at the start of their assessment resulted in higher final accuracy than when providers made a preliminary diagnosis before viewing AI results, and than either the provider or AI alone.^8^ This was true even with moderate AI model accuracy.^8^ It would be interesting to know whether these trends hold in primary care settings with larger sets of potential diagnoses and when an AI model was not trained with all possible outcomes as in Zeltzer et al.^2^

Zeltzer et al's^2^ evaluation is intentionally restricted to a relatively stable state of technology. Although a useful starting point, AI performance shift can occur because of changes in the population accessing the technology (data drift), infrastructure and design, end-user behavior (eg, intake response patterns changing input data, or augmented clinical decision-making skill changes), or model retraining.^9^ Identifying and monitoring impacts on patients, providers, and systems over time and with continued use is a multidisciplinary challenge that will require broad and deep investigations.

### Social Queries

The analytic sample in Zeltzer et al^2^ included 17.4% (n=102,059) of the 586,819 people who started the AI-based virtual primary care intake.^2^ Why did 96,114 people drop out partway through the AI-based intake procedure? What happened to the 372,910 people who finished the AI-based interview and chose to not receive a provider diagnosis after seeing the AI differential diagnosis? Are safety protocols in place or needed for people that decline human interaction but for whom the AI model predicts a condition in need of urgent attention (eg, suicidal ideation)? Demographic characteristics were not reported for these groups nor for those excluded from the analytic sample because of complex or challenging diagnoses (multiple or nonspecific ICD-10 codes).^2^

Sociotechnical analysis is needed to investigate bias, equity, and provider and patient experiences throughout the entire AI-first clinical paradigm. Whether and how social determinants of health are captured and used needs to be considered as social determinants have been found to influence AI access, uptake, and effectiveness.^10^ Critical, comprehensive study into how, when, and for whom an AI-first virtual primary care paradigm does and does not work well is necessary for equitable scale-up and population health impact.

Zeltzer et al^2^ provide novel evidence around a piece of the complicated puzzle of AI diagnostic support for virtual primary care, motivating further sociotechnical evaluations. There is great potential for AI to support increased efficiency, effectiveness, and equity of primary care; there is also a risk of opposite, harmful impacts.^1^ Given the diversity in potential impacts of AI, mixed methods evaluations focused on specific use cases may be a valuable next step toward understanding those altogether not so uncommon scenarios in primary care.

## Potential Competing Interests

The authors report no competing interests.
